# Surgical waiting lists and queue management in a Brazilian tertiary public hospital

**DOI:** 10.1186/s12913-024-10735-4

**Published:** 2024-03-06

**Authors:** Antonio Pazin-Filho, Maria Eulália Lessa do Valle Dallora, Tonicarlo Rodrigues Velasco, Roberto de Oliveira Cardoso dos Santos, Gustavo Jardim Volpe, Diego Marques Moroço, Danilo Arruda de Souza, Claudia Marques Canabrava, Luis Vicente Garcia, Edwaldo Edner Joviliano, Benedito Carlos Maciel

**Affiliations:** 1 Ribeirão Preto Medical School, University of São Paulo, Ribeirao Preto, São Paulo Brazil; 2 Clinical Hospital - Ribeirão Preto Medical School, University of São Paulo, Ribeirao Preto, São Paulo Brazil

**Keywords:** Hospital administration, Surgery department, Hospital, Waiting lists, Operating room information systems, Universal access to health care services, Barriers to access of health services, Elective surgical procedures, Health management, Decision modeling, Summed

## Abstract

**Background:**

Centralized management of queues helps to reduce the surgical waiting time in the publicly funded healthcare system, but this is not a reality in the Brazilian Unified Healthcare System (BUHS). We describe the implementation of the “Patients with Surgical Indication” (PSI) in a Brazilian public tertiary hospital, the impact on waiting time, and its use in rationing oncological surgeries during the COVID-19 Pandemic.

**Methods:**

Retrospective observational study of elective surgical requests (2016–2022) in a Brazilian general, public, tertiary university hospital. We recovered information regarding the inflows (indications), outflows and their reasons, the number of patients, and waiting time in queue.

**Results:**

We enrolled 82,844 indications in the PSI (2016–2022). The waiting time (median and interquartile range) in days decreased from 98(48;168) in 2016 to 14(3;152) in 2022 (*p* < 0.01). The same occurred with the backlog that ranged from 6,884 in 2016 to 844 in 2022 (*p* < 001). During the Pandemic, there was a reduction in the number of non-oncological surgeries per month (95% confidence interval) of -10.9(-18.0;-3.8) during Phase I (January 2019-March 2020), maintenance in Phase II (April 2020-August 2021) 0.1(-10.0;10.4) and increment in Phase III (September 2021-December 2022) of 23.0(15.3;30.8). In the oncological conditions, these numbers were 0.6(-2.1;3.3) for Phase I, an increase of 3.2(0.7;5.6) in Phase II and 3.9(1,4;6,4) in Phase III.

**Conclusion:**

Implementing a centralized list of surgical indications and developing queue management principles proved feasible, with effective rationing. It unprecedentedly demonstrated the decrease in the median waiting time in Brazil.

## Background

Hospital surgical production is a complex process involving several stages, from the organization of surgical teams, the availability of rooms and permanent and consumable material, and the organization of other care resources, such as intensive care units (ICU). In addition, the complete pathway involves preoperative steps (pre-anesthetic consultation and surgical preparation) and postoperative care that vary according to the severity of the disease, size and nature of the surgical intervention, medical specialty involved, and patient conditions, whether related to health (comorbidities and clinical-functional vulnerability indices), locoregional residence, or socioeconomic factors.

Despite the complexity of the process, surgical procedures imply higher remuneration and a higher revenue volume for hospital entities. It also means less variability in production for elective procedures and better control of direct and indirect costs. Elective surgical procedures precisely indicated and timely performed mitigate costs to health systems, decreasing searches for the emergency department. Furthermore, the surgical waiting time in Brazil is attracting the public’s attention, demanding health policy to improve transparency, management of resources, and healthcare governance. Managers should be attentive to all the previous statements to guarantee the quantity and quality of the production of elective surgeries.

Commonly, surgical teams are responsible for managing the flow of elective indication lists. The fragmentation of these lists in different specialties makes it difficult for hospital managers to have a global view. Moreover, centralizing the lists helps overcome this deficiency, including at the state or national level in countries with a public health system, such as the United Kingdom, Canada, Australia, and Spain [1–7]. Recently (2023), Brazil has had a national interest in managing surgical queues, including establishing the National Program to Reduce Queues for Elective Surgery, Complementary Exams, and Specialized Consultations [8].

Lists and surgical queues, considered synonyms, are rationing mechanisms in the absence of a market or the event of scarcity of resources: unmet needs, inefficient management, or low productivity results in longer waiting times. For now, there are discussions about the poor distribution of hospital resources, little or no programming based on health needs, low management capacity, and questionable performance in the Brazilian Unified Health System (BUHS) [9, 10].

However, one should distinguish the terms list and queue. Queuing theory supports this problematization in Little’s Law, whose mathematical formula is “L = λ.W” where ‘L’ indicates the number of cases in a list, ‘λ’ the arrival rate (entry speed of cases patients on the list), and ‘W’ the average waiting time for production or delivery of the product (in the case under discussion, surgeries). Thus, the waiting list would be one of the components of the surgical queue. Managing the lists would be necessary to minimize the waiting time, the incoming flow of patients, and the production of services. The health system would achieve efficiency when acceptable waiting times were reached [10].

This article describes the implementation process of a centralized list system for surgical indications and queue management in a Brazilian general, public, and tertiary university hospital. It deals with a seven-year experience with solid results that helped the hospital institution face the COVID-19 Pandemic. Since no data has been published describing devices, actions, and instruments by the federal government regarding the BUHS Program to reduce the number of elective surgeries, this study is a timely and relevant contribution.

## Methods

We conducted a retrospective observational study on the database of surgical requests performed at Hospital das Clínicas da Faculdade de Medicina de Ribeirão Preto (HCFMRP-USP) between 2016 and 2022. The HCFMRP-USP is a state institution that is a tertiary reference for a population of 4.5 million inhabitants in the northeast of the State of São Paulo and the university education and teaching field. The HCFMRP-USP performs highly complex procedures in different surgical areas, being a state and national reference. The institution has more than 50 specialties and several highly complex qualifications, with 600 beds, 14 operating rooms, and an annual average of 13,930 surgeries (2016–2019).

Until 2015, the surgical teams managed indications in dedicated lists with restricted access to its members, not including the institution administrations that only evaluated production. As of 2016, the institution unified waiting lists for surgery by specialty in an Electronic Medical Record (EMR) module. The EMR is an in-housing development that allows the institution to update and customize it. The surgical queue module is designated “Patients with Surgical Indication” (PSI). After unifying all surgical lists into the system, a team could only schedule a patient for a surgical procedure if included in the PSI. Mandatory enrollment in the PSI ensured adherence to institutional guidelines. It allowed the centralized management of this list, removing requests that no longer had an indication (change of conduct or death) or those already operated on. In this process, we developed queue management mechanisms, with rules for inserting and removing requests and guidelines for the systematic review of the queue management process, which became manuals available to the system user. We present the PSI’s management principles in Table 1.

**Table 1:** Patients with surgery indication (PSI) - system management principles

PIC Management Principles		
***1***	***Waiting list***	
		a) Mandatory - A patient can only have a surgical appointment if registered in the PSI system. b) Inclusion - Registration of surgical indications in the system by residents or attending physicians. c) Validation - Conduct verification process and prioritization carried out by the physician responsible for the specialty (coordinator or preceptor) who becomes the co-manager of the queue with the Administration.
***2***	***Surgical Queue Management***	
		a) Co-management - Responsibility for controlling indications rests with the person in charge of the surgical specialty. The Administration does not interfere with the technical criteria or the order of surgical indication. It is up to the Administration to monitor the indicators of each specialty, conducting periodic discussions for necessary adjustments to guarantee the feasibility of the inserted demands. b) Monitoring indicators - Each specialty has access to its management level (patients indicated by the specialty), with information relevant to the indication date, the validity of the preoperative evaluation, etc. This mitigates comparisons or misuse of sensitive data across specialties. Based on the indicators, the validating physician can reposition the patient in the queue, advancing or delaying as necessary. Management has an overview and a view of each specialty for decision-making. c) Traceability - The system documents every change made to the list. There is space to insert observations regarding the patient’s condition or logistical conditions (material availability, for example) that justify the change. If the patient has the indication removed, one should document the reason. d) Administrative use for offer management - Although the Administration does not interfere in the management of the queue from a technical point of view (pertinence of the indication and prioritization criteria), it is co-manager of the queue by guaranteeing resources that make it possible to carry out what was scheduled and by using the median waiting time for each specialty to offer vacancies to the SUS manager, managing the external demand. As a general rule, if the specialty has more than three times its available surgical capacity already scheduled for the next six months, the offer is suspended.

The enrollment of patients in the system begins with the registration of the surgical request by the responsible medical professional (Applicant). By inserting patient identification in the solicitation form, demographic data are retrieved (gender, age, address), and the PSI provides information if the patient has already had a previous indication, has already been completed (surgery performed or indication suspended), or remains valid. If there is a pending indication, the requesting physician can decide whether to include the surgical procedure. Subsequently, the PSI requests data regarding the type of procedure indicated– International Classification of Diseases (ICD) 10 code, necessary resources (image intensifier, special prostheses, etc.), and whether there is a need for postoperative care in the ICU. The applicant can assign immediate priority or a scale from 1 (highest) to 4 (lowest) for elective conditions. The “Immediate” condition refers only to conditions scheduled as elective, which became urgent during the waiting list. The institution deals with urgent referrals in another building unit of the HCFMRP-USP, located seven kilometers from the main structure. After insertion, there is a need for validation by the person in charge of the specialty (Validator), making him the queue manager in that specialty and co-responsible for managing the queues at the institution with the Administration.

The PSI has hierarchical indicators by prioritization level according to surgical specialty, allowing the Validator and the Administration to consult the queue condition by any variable of the registration form. It presents a queue organized by order of inclusion and prioritization by default. The validator can change the patient’s order in the queue or remove the indication. The Administration can consult indicators and hold periodic meetings for adjustments with the queue manager (Validator) in their respective specialties to guarantee that the waiting time for surgery remains within the limit of up to 3 times the surgical capacity of each specialty. This goal resulted from the experience of 7 years in managing elective surgical queues at HCFMRP-USP. Furthermore, the PSI automatically deletes any request if the patient dies while waiting for surgery.

We merged PSI information with other EMR databases - surgical production data (surgery date, and reason for cancellation) and in-hospital outcomes (discharge, transfer, or death). With this unified database, we obtained information regarding inflows (indications), outflows and their reasons, the number of patients, and waiting time in the queue. It was possible to evaluate the behavior of these variables during the Pandemic.

In summary, our Operational Definition of Variables are the following: (A) Indicated - number of patients in list in the correspondent year; for the year 2016, considering that was the first year of the software implementation, it includes the patients from the previous years as well; (B) Reason for list withdrawal classified by reason– operated on (surgery in the same year), patient (did not have conditions for the procedure on the day of surgery), team (interventions by the team responsible for cleaning the list), administrative (central administration interventions), duplicated (patient had more than one indication in the queue for the same procedure), previously operated on (without having been withdrawn in the relevant year) and death; (C) Backlog corresponds to the difference between those indicated and those who left the queue in the corresponding year; (D) Number of indications waiting in the list corresponds to the sum of the backlog of the previous year with those indicated in the current year minus the patients withdrawing from the queue in that year; (E) Waiting time corresponds to the median (interquartile range) in days for those in the list in the corresponding year. We calculated the average waiting time in the queue as the difference between the indication date (inclusion in the PSI) and the exit date.

We used STATA 15 for data analysis and figure construction. We used the Kruskall-Wallis and Mann-Kendal test for the tendency to compare the waiting times and backlogs from 2016 up to 2022. We used interrupt time series with Newey-West regression to analyze the impact of rationing oncology surgeries during the COVID-19 Pandemic. The level of significance for all tests was 0.05.

The HCFMRP-USP´s Research Ethics Committee approved the protocol (CAE 57670122.1.0000.5440). The requirement for informed consent was waived by the Ethics Committee of HCFMRP-USP´s Research Ethics Committee because of the retrospective nature of the study.

## Results

We enrolled 82,844 requests in the PSI between 2016 and 2022–8,830 (10.6%) before 2016 and added to the PSI when it started.

Table 2 presents the results of the inflow, outflow, and number in the list. The number in the list resulted from the previous year’s backlog to those requested in the current year minus those leaving the queue. For example, it started in 2016 with 8,830 requests, of which 1,946 were withdrawn from the list (88% due to previously performed surgery). The backlog consisted of 6,884, which, at first, corresponded to the number of patients in the list. In 2017, the number of people in the queue was added to the indications (16,469) and subtracted from the exits (16,785; 12,846 operated), leaving 6,568 people in the queue. This process takes place progressively.

**Table 2:** Number of surgical indications (A), motives of list withdrawal (B), backlog (C), number of indications waiting in the list (D), and median (interquartile range) for the waiting time in days (E) according to the year. In B, we present the percentage of the reasons for list withdrawal

		2016		2017		2018		2019		2020		2021		2022		*p*
Indicated(A)		8830		16,469		17,303		15,110		8376		10,529		8534		< 0.05
List withdrawal (B)	Operated on	1714	88%	12,846	77%	13,443	83%	12,280	79%	7254	85%	8969	73%	6489	84%	0.677
	Patient	31	2%	1165	7%	872	5%	945	6%	287	3%	849	7%	352	5%	
	Team	37	2%	1565	9%	1027	6%	1199	8%	500	6%	1475	12%	532	7%	
	Administrative	1	0%	121	1%	183	1%	186	1%	51	1%	97	1%	25	0%	
	Duplicated	20	1%	236	1%	252	2%	230	1%	107	1%	266	2%	82	1%	
	Previously operated on	59	3%	586	3%	134	1%	421	3%	98	1%	255	2%	25	0%	
	Death	84	4%	266	2%	296	2%	284	2%	258	3%	350	3%	185	2%	
	Subtotal	1946		16,785		16,207		15,545		8555		12,261		7690		< 0.05
Backlog (C = A-B)		6884		-316		1096		-435		-179		-1732		844		
Number of indications waiting in the list (D = C (previous year) + C (Current year)		6884		6568		7664		7229		7050		5318		6162		< 0.05
Waiting time in days (E) - median (interquartile range)		98(48;168)		58(6;195)		41(5;144)		44(4;175)		10(2;96)		13(3;393)		14(3;152)		< 0.05

(A) Indicated - number of patients in list in the correspondent year; for the year 2016, considering that was the first year of the software implementation, it includes the patients from the previous years as well; (B) Reason for list withdrawal classified by reason– operated on (surgery in the same year), patient (did not have conditions for the procedure on the day of surgery), team (interventions by the team responsible for cleaning the list), administrative (central administration interventions), duplicated (patient had more than one indication in the queue for the same procedure), previously operated on (without having been withdrawn in the relevant year) and death; (C) Backlog corresponds to the difference between those indicated and those who left the queue in the corresponding year; (D) Number of indications waiting in the list corresponds to the sum of the backlog of the previous year with those indicated in the current year minus the patients withdrawing from the queue in that year; (E) Waiting time corresponds to the median (interquartile range) in days for those in the list in the corresponding year

The waiting time in days (median and interquartile range) in the queue decreased from 98(48;168) days in 2016 up to 14(3;152) days in 2022(*p* < 0.01) (Table 2). The centralization and list management allowed for a negative backlog in four of the seven years evaluated, three consecutive (2019–2021). In 2018, there was a positive backlog. However, from that year onwards, the offer of vacancies for surgery became restricted by the waiting time, exemplifying the use of the list to manage the offer to the BUHS manager. In 2022, there was an increase in the number of remaining patients due to the need for screening for a surgery campaign promoted by the São Paulo State Health Department. Looking at the 75% percentile of the interquartile range for the waiting time, we see the same trend throughout the analyzed period, ranging from around 3 to 6 months, except for 2021 (393 days; 13 months).

**Fig. 1:**
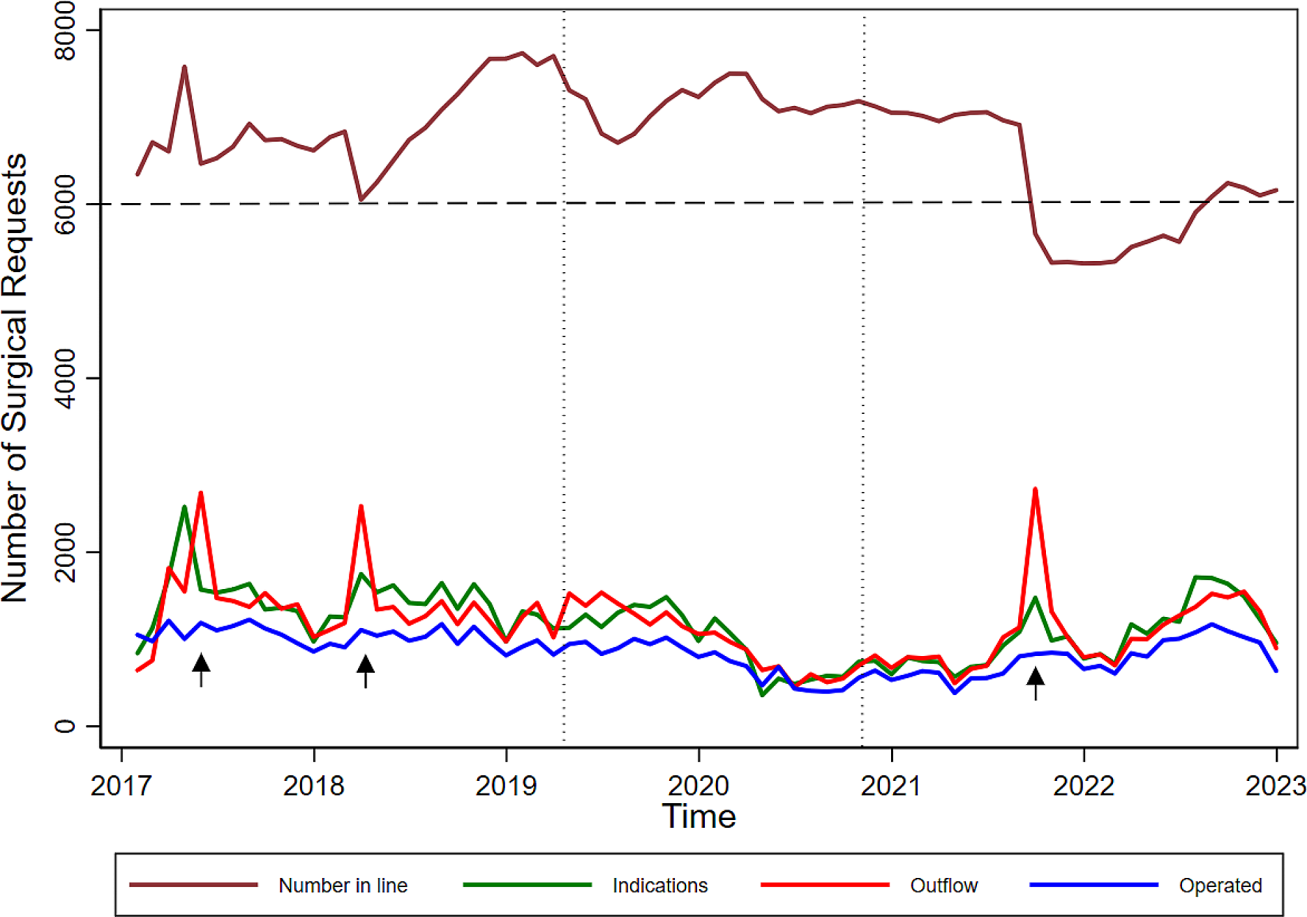
Distribution of the number of patients in the queue, indications, discharges (withdrawing from the queue), and operated on as a function of time. The horizontal dashed line represents the limit of patients in the institution’s queue as a condition for the queue management process. The horizontal dashed lines represent the period of the COVID-19 Pandemic. The arrows represent systemic interventions by the Administration to promote the review of the queues by the managers of the specialties

Table 2 details the outflow reasons, mainly due to the proposed surgery (ranging from 73 to 88%). The reasons related to the patient (ranging from 2 to 7%) and the team (ranging from 2 to 12%) reached the highest percentages after the surgical procedure conclusion as planned. The proportion of patients excluded for various reasons tended to remain constant. Figure 1 illustrates three list sanitation promotion periods promoted by the Administration that increased the proportion of removal from the list without surgery.

**Fig. 2:**
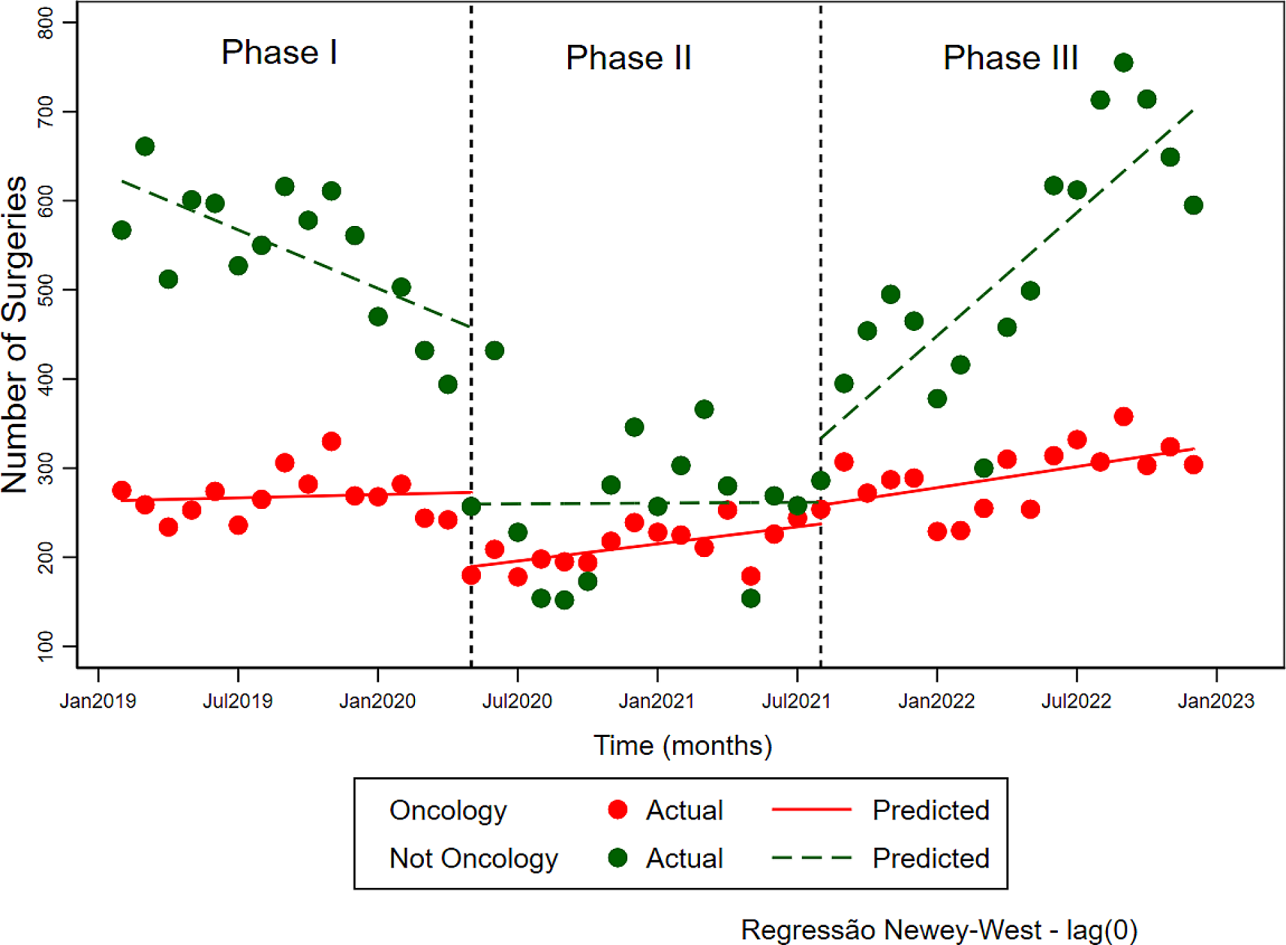
Number of patients operated on for oncological and non-oncological conditions as a function of time and phases of the COVID-19 Pandemic. Horizontal dashed lines demarcate the pandemic phases– I (first wave), II (second wave– period of a significant reduction in surgical movement), and III (post-pandemic recovery). The solid lines (red) and dashed lines (green) are the results predicted by the Newey-West model used in the analysis of interrupted time series

The management of the surgical list allowed the institution to ration oncological surgeries during the most critical period of the COVID-19 Pandemic. Figure 2 illustrates surgical production in the first wave (Phase I– January 2019-March 2020), in the second wave (Phase II– April 2020-July 2021), and in the post-pandemic recovery (Phase III– August 2021-December 2022). When we observe the behavior of non-oncological surgeries, there was a reduction in the number of surgeries per month (95% confidence interval) of the order of -10.9(-18.0;-3.8) during Phase I, maintenance of the number of surgeries in Phase II 0.1(-10.0;10.4) and increment in Phase III 23.0(15.3;30.8). In the oncological conditions, these numbers were 0.6(-2.1;3.3) for Phase I, an increase of 3.2(0.7;5.6) in Phase II and 3.9(1,4;6,4) in Phase III (Fig. 2).

## Discussion

The central hypothesis for surgical queue generation is the imbalance between demand and capacity. Nevertheless, observation of the behavior of queues raises other hypotheses. If the demand were continuously greater than the capacity, the queues would grow exponentially, but one observes stability when a certain number of patients is reached in most queues [11]. So, other hypotheses were proposed, such as the “Backlog” Theory, a period in which a marked imbalance between demand and capacity exceeded expectations. This theory would support the proposal of corrective measures to adjust the waiting time in queues with a transient increase in capacity [11]. In the present work, we observed the behavior of the backlog as described and the importance of interventions to limit its growth and adjust the waiting time in the queue.

Loss of surgical schedules due to patient non-attendance, lack of clinical conditions for surgery, or lack of ICU or ward beds for post operative care could also be confounders [12]. The factors arising from the patients were around 3 to 7% in the present study, consistent with what was observed by Hovlid et al. [13]. We reduced the impact of the lack of intensive care and ward beds through the intervention of the institution’s Internal Regulation Nucleus (IRN) [14].

The co-management of the queue with the elaborated criteria allowed the gradual reduction of the refractoriness of surgical specialties to list centralization and queue management. Teams can use long queues as an argument to get more appointments for their specialty [2]. Procedures considered less challenging or less “urgent” are postponed by list managers [1]. This phenomenon became known as “the inverse burden law of specialists” [11]. We minimized these factors, helping to obtain the resources for the procedures for those requests in the centralized list, and the specialties began to appreciate the proactive management mechanisms implemented by the Administration.

Although surgical queues have a negative connotation, they ensure transparency of the system’s liability to solve and enable planning interventions. Although the disproportion between production capacity and demand recognition is variable between healthcare systems and regions of the same system, as is the case of the BUHS, all are susceptible to rationing periods. We demonstrated how effective prioritization helped manage oncological cases during the COVID-19 Pandemic. Another advantage of surgical queues is scheduling according to the availability of resources, reducing the loss of surgical schedules due to lack of material, and better scheduling to ensure teaching. The opposition to these arguments is that a long surgical queue could cause harm to patients, in addition to anxiety and uncertainty about when to schedule the procedure.

The centralized queue management observed in this study showed an objective reduction in waiting time. Although the waiting time may be adequate for a given situation, the perception of time and acceptance of the waiting condition can generate dissatisfaction on the part of patients, leading to the search for internal or external administrative interventions or the search for the media. With the advent of information technology and social networks, some institutions seek to make the waiting process transparent by making the lists available on their websites. However, there is evidence that comparing queues made available by the institutions is difficult to interpret, as the management is peculiar to each institution [15]. Although data transparency is desirable, this process must consider Brazil’s recently enforced General Data Protection Law.

Queue management must ensure the removal from the list of already operated patients or who no longer have an indication [16]. The internal audit has a proactive role, such as confirming the attendance of patients on the day of surgery, assessing whether the surgeries performed involved removing the patient from the queue and relocating the surgical grid to avoid loss of schedules in case of unexpected cancellations.

In addition to these already implemented activities, there are other possibilities, such as establishing a pattern of acceptable queuing for each specialty and using artificial intelligence to identify predictors of this condition, allowing preventive action. Levy et al. point out the need to modify the paradigm of a list for managing waiting time to a tool that accompanies the preparation of patients for the surgical procedure [17]. It is noteworthy that a centralized queue management mechanism must have execution tools represented by the IRN or case managers to guarantee the interventions.

The definition of an adequate waiting time for each type of surgery is still being determined. In the original conceptions of the British National Healthcare System, up to one year was accepted [2]. The waiting time between the patient’s referral and the beginning of his treatment for elective circumstances is 18 months. For Canada, there is a recommendation of 4 to 17 weeks [4]. The waiting time in the queue can and should be variable for each condition. With the development of waiting lists, several specialties and their societies recommend an acceptable average waiting time [18, 19]. Ballini et al. performed a systematic review to evaluate different strategies to reduce the waiting time for surgical procedures. The high heterogeneity of the collected data prevented the performance of a meta-analysis. However, the conclusions presented may help compare the individual conditions of each situation, even though they do not allow generalization [20]. However, there is no data for all types of surgery and no established standard for the Brazilian reality.

The COVID-19 Pandemic had overwhelming implications for the world and Brazil, with a significant increase in directly related mortality [21]. In addition, the redirection of the hospitals’ capacity to face the enormous challenge reduced the installed surgical capacity. Undoubtedly, the main reason was the need for health professionals to compose additional intensive care teams. Still, there was also fear of patients undergoing elective procedures, amplified by the association of COVID-19 with higher perioperative mortality [22].

Regardless of the most significant factor, the reduction in surgical capacity increased the backlog of surgeries [23]. Several institutions have described the most significant impact on surgeries whose delay implied less risk of life or loss of function [24–26]. Among these initiatives, the implementation of surgical lists or priority changes are cited as strategies [27–29]. We confirmed the prioritization of oncological surgeries as an efficient strategy when the institution faced a decrease in its general surgical capacity to cover all the demands of the Pandemic.

This work is limited because external referral urgent surgeries were managed in another hospital, which does not occur in other services [30]. However, during the Pandemic, several elective surgeries on hold became emergencies, and there was a need to balance the daily demand.

The unified management of the surgical queue at a tertiary public hospital proved feasible and required the development of co-management principles. These principles allowed for reducing the median waiting time in the queue and prioritizing patients to achieve institutional goals during the COVID-19 Pandemic.

## Conclusions

The present study is the first to describe a centralized list and management of the surgical waiting queue in Brazil. The work addresses the strategies for unifying and centralizing the queue, the principles for its management, and its use so that the institution can successfully adapt to the challenges of the COVID-19 pandemic. The guidelines developed open the possibility of replication for other services and may contribute to national centralization, as observed in other countries.

Although the results are specific to the institution, they certify that the centralization of the list and queue management are essential strategies for managing surgical teams, internal auditing, and improving the performance of hospital institutions. Efforts to implement the list and the queue simultaneously affected the organization of resources and the provision and programming of services to the BUHS manager, improving institutional results and, consequently, the region. In this sense, its constancy and improvement actively contribute to ensuring equity in the BUHS.

## Data Availability

The datasets generated and/or analyzed during the current study are not publicly available due to national regulations but are available from the corresponding author on reasonable request.

## Abbreviations

BUHS: Brazilian Unified Health System
COVID: 19-Coronavirus Disease 19
EMR: Electronic Medical Record
HCFMRP: USP-Hospital das Clínicas da Faculdade de Medicina de Ribeirão Preto
ICD: International Classification of Diseases
ICU: Intensive Care Unit
IRN: Internal Regulation Nucleus
PSI: “Patients with Surgical Indication” software
